# Interdisciplinary strategies for establishing a trusting relation as a pre-requisite for existential conversations in palliative care: a grounded theory study

**DOI:** 10.1186/s12904-025-01681-x

**Published:** 2025-02-19

**Authors:** Annica Lagerin, Christina Melin-Johansson, Bodil Holmberg, Tove Godskesen, Elin Hjorth, Lena Junehag, Carina Lundh Hagelin, Anneli Ozanne, Johan Sundelöf, Camilla Udo

**Affiliations:** 1 Department of Health Care Sciences, Marie Cederschiöld University, Stockholm, Sweden; 2 Department of Health Care Sciences/Nursing, Mid Sweden University, Kunskapens väg 8, Östersund, 831 25 Sweden; 3 Department of Health and Caring Sciences, Linnaeus University, Växjö, Sweden; 4 Faculty of Nursing and Health Sciences, Nord University, Bodø, Norway; 5 Centre for Research Ethics & Bioethics, Department of Public Health and Caring Sciences, Uppsala, Sweden; 6 Department of Neurobiology, Care Sciences and Society, Division of Nursing, Karolinska Institutet, Stockholm, Sweden; 7 Institute of Health and Care Sciences, Gothenburg University, Sahlgrenska Academy, Gothenburg, Sweden; 8 Department of Neurology, Sahlgrenska University Hospital, Gothenburg, Sweden; 9 Department of Public Health and Caring Sciences, Uppsala University, Uppsala, Sweden; 10 Department of Health and Welfare, Dalarna University, Falun, Sweden

**Keywords:** Communication, End-of-life, Existential, Healthcare personnel, Interdisciplinary, Palliative care, Presence, Qualitative methods

## Abstract

**Background:**

Communication is central to implementing palliative care (PC) and effective interdisciplinary team functioning. Communication about existential issues is often urgent in PC, yet interdisciplinary teams frequently lack the time and education to meet these communication needs. Thus, more knowledge of existential conversations in different PC contexts is required.

**Aim:**

This study aimed to gain an in-depth understanding of healthcare professionals’ (HCPs) experiences of existential conversations with patients with PC needs and their next-of-kin by generating a theoretical model.

**Method:**

Seven focus-group interviews that included 26 HCPs who worked with PC patients in different care settings were carried out in 2020 and 2022. The grounded theory method was used to analyse and compare data from the interview transcripts.

**Results:**

The HCPs’ primary concern in daily work was *establishing a trusting relationship*, a prerequisite for enabling existential conversations with a person with PC needs and/or their next-of-kin. The main concern was characterised by the core category *maintaining presence* and four categories describing interdisciplinary strategies that the HCPs used to achieve a trusting relationship and enable existential conversations in the late phase of life. Several potential barriers also hindered existential conversations. The theoretical model ‘meaningful existential conversations in PC’ was constructed.

**Conclusions:**

The interdisciplinary strategies used to establish existential conversations, the potential barriers to these conversations and the model we present can be used as a basis for reflection in professional collaborative learning in PC, as a tool for teachers in educational PC programmes and as a guide for HCPs in PC.

**Supplementary Information:**

The online version contains supplementary material available at 10.1186/s12904-025-01681-x.

## Background

When living with a life-threatening illness, physical losses are often experienced in combination with a loss of meaning, hope and a general loss of control over life [1]. There may also be a fear of dying [2]. Therefore, healthcare professionals (HCPs) need to be prepared to support severely ill patients and their next-of-kin in the form of conversations [3]. According to the World Health Organization’s (WHO) definition of palliative care (PC), the healthcare system should offer support to these patients and their families to help them cope with the patient’s’ illness and the families’ bereavement [4].

In Sweden, approximately 90.000 adults die every year, and nearly 80 per cent die from slowly progressive diseases such as cancer, cardiovascular disease or dementia. This suggests that many individuals have, at some point, made contact with the healthcare system where planning and decisions have been made concerning their care. In Sweden, PC should be provided equally, regardless of the healthcare context (e.g. in nursing homes, as primary care and by specialised homecare teams). Homecare teams should also provide additional support to HCPs in the context of primary care and in nursing homes while provisioning PC [5]. However, access to PC is unevenly distributed across the country, and in some localities, no specialised teams are available [6].

Within all healthcare contexts, communication is central to implementing PC and effective interdisciplinary team functioning [7]. A palliative interdisciplinary team often includes physicians, registered nurses (RNs), assistant nurses (ANs), physiotherapists (PTs), occupational therapists (OTs), and dietitians. Other related professionals may include spiritual carers, psychologists and hospital social workers who support religious, existential and/or psychosocial well-being [8, 9]. To emphasise the importance of ethical competence for quality patient care, particularly in PC, a combination of care ethics, clinical proficiency, relational skills and effective communication is essential [10–12]. 

HCPs from diverse disciplines need to cooperate [10]. In Sweden, PC teams are usually led by physicians and RNs in cooperation with related HCPs [7]. They are expected to assess not only the patient’s physical needs but also their psychological, social, spiritual and existential ones and to regard patient suffering as multidimensional [4]. Good communication skills and the ability to listen attentively and with compassion facilitate these conversations [11]. A previous study found that PTs and OTs generally hold positive beliefs about advanced care planning and end-of-life conversations, but common barriers to these discussions include a lack of confidence, perceived patient and family reluctance, organisational challenges and a lack of role clarity [12]. Moreover, nursing home professionals such as ANs and care assistants are task-oriented and often lack the time to talk to patients and their next-of-kin [13]. Similarly, RNs in nursing homes are responsible for many patients and primarily function as consultants, thus they also lack the time to talk to individual patients and next-of-kin [14]. Another aspect that may prevent HCPs from holding conversations with severely ill patients and their next-of-kin is an inherent feeling of inadequacy paired with a fear of unintentionally uncovering ideas that will make the patient or next-of-kin pose questions that the professionals are unable to respond to [11].

In the Swedish National Guidelines for PC, the importance of various forms of conversations in the context of PC is emphasised, e.g. end-of-life discussions, advanced care planning, as well as supportive and existential conversations. These discussions may be planned or can occur spontaneously during care interventions. The goals are, among others, to improve care planning, support the patient’s quality of life, relieve symptoms, build trust, and– in accordance with Swedish laws– enable patient participation in their own care [15].

Not all severely ill patients and their next-of-kin have access to HCPs with professional knowledge and training in existential conversations about life and death. This is problematic as the aim of PC is to prevent suffering in all severely ill patients, irrespective of their disease or location [16]. Moreover, to achieve that aim, communication is needed. Hence, we need to increase our knowledge about existential conversations in different PC contexts to support equal access to high-quality PC. Therefore, to learn more about how communication is conducted and handled in PC by different HCPs, we investigated their main concerns and strategies regarding existential conversations.

## Aim

To gain an in-depth understanding of HCPs’ experiences of existential conversations with patients with PC needs and their next-of-kin by generating a theoretical model.

## Materials and methods

### Study design

This qualitative study was part of a larger, mixed-method research project, “Talk for life– conversations in palliative care”, that included different professions from several care contexts in general and specialised PC units in rural and urban areas in Sweden. The classic grounded theory method (GTM) [17] was chosen to generate a theoretical model to explain underlying patterns that emerge when HCPs have existential conversations with patients in PC and what strategies they use to manage these conversations. Focus-group interviews (FGs) [18] were the primary data source and included HCPs from different care settings. Since FG has the potential to provide rich data including discussions of different views on the same phenomena, FG were chosen as a data collection method. GTM is a qualitative research methodology focused on developing theories from the data rather than testing pre-existing hypotheses. The process involves constant comparative analyses to uncover patterns, concepts and categories within the data, leading to the new theoretical insights. In this study, data from FGs in 2020 was used to develop and deepen the interview questions in the data collection 2022.

### Setting and participants

Data for this study was collected in 2020 (phase 1) and 2022 (phase 2). To obtain rich material, an effort was made to collect data from different care settings, and HCPs were identified using purposeful sampling. RNs, ANs, CAs, OTs and PTs who had worked in their profession for at least six months were included (Table 1). HCPs who conduct professional counselling, such as social workers, psychologists and priests, were excluded. Since focus was on existential conversations that could spontaneously arise, professions who worked closely to the patients, meeting them on daily basis, were eligible. Study participants worked in rural and urban areas in two regions of Sweden. The HCPs were invited to participate in the study by managers at patient care units who received written and oral study information from the researchers. In 2020, open sampling was used to include HCPs from one hospice unit and one specialised PC unit in three FGs (2–4 participants/group). We used theoretical sampling according to GTM [19]. When questions emerged from an analysis of the data from 2020, the authors developed an interview guide to focus on new questions and conducted new FGs in 2022. In 2022, four additional FGs (4 participants/group) in three nursing homes were conducted. A total of 26 HCPs provided written consent and participated in FGs performed at their workplaces. To achieve a trusting environment for the participants to freely share their experiences, each FG were introduced by discussing the study, opening for any reflections or questions, followed by presentation of everyone in the room and with some small talk before starting the FG. The authors led the FGs in pairs, where one conducted the interview while the other observed the interaction between the participants [18]. Each FG lasted 45–90 min and was audio recorded and transcribed verbatim.

**Table 1:** Demographic and clinical information

Variables	*N* = 22
**Age**	
Median Min-max, years	40 28–63
**Gender**	
Female Male	19 3
**Profession**	
Assistant nurses (ANs) Registered nurses (RNs) Care assistants (CAs) Physiotherapist (PTs) Occupational therapist (OTs)	11 5 2 2 2
**Care settings**	
Nursing homes Specialist palliative care Hospice	16 3 3
**Work experience at the units**	
> 1 year 1–5 years 6–10 years < 10 years	1 7 10 4

### Data collection– phase 1

The FGs conducted in 2020 included ANs, RNs, PTs and OTs in one hospice and one specialised PC ward in an urban area in one region of Sweden. An interview guide (see supplementary file) with open-ended questions was developed for the present study to understand what was important from the participant’s viewpoint. Open-ended questions in this phase included: ‘Can you tell me what conversations arise when you are caring for patients and when meeting their next-of-kin?’ or ‘Can you describe what made the conversation good or less good?’ In the initial phase of the analysis, codes and concepts were identified and compared with each other, which resulted in five preliminary categories: *conversations about death*, *thoughts about the time left*, *not ready to talk about existential issues and imminent death*, *fear of talking to children* and *next-of-kin wants to decide*.

### Data collection– phase 2

In 2022, FGs were conducted at the workplace of CAs, ANs and RNs in four wards at three nursing homes in rural and urban areas in two regions of Sweden. Our rationale for collecting data from CAs, ANs and RNs in nursing homes was to compare and saturate emerging concepts and to gain conceptual clarity between the data in phases 1 and 2. The interview questions were based on leads from the preliminary categories in the FGs from 2020 (phase 1). Thus, the interview questions became more focused and in-depth. For example: ‘Can you tell me how you communicate with a patient regarding their thoughts about death?’, ‘Can you describe how you respond to patients’ existential questions?’ and ‘Can you tell me how you respond to next-of-kin who don´t accept the situation?’ In accordance with GTM [17], the participants were chosen for their theoretical relevance to further develop the categories identified in the FGs from 2020 (phase 1).

### Analysis

In line with classic GTM [17], the analysis process began during the interview stage. It was initiated by conducting open coding on each transcript; subsequently, four authors (AL, BH, CMJ, CU) individually read all seven interview transcripts line by line. In phase 1, data collected in 2020 were analysed using open coding, and the codes were grouped into five preliminary categories while remaining open to the latent pattern. Memos were documented during the analysis to record reflections on key concerns and concepts and supported the process between data collection and the writing procedure [17]. In phase 2, data collected in 2022 were analysed, and focused coding was used to deepen and broaden the preliminary categories from phase 1 described in the result. The researchers collected data until they estimated that saturation was achieved, i.e. no new concerns or concepts appeared [17], and the developing main category was formed around the main concern, *establishing a trusting relationship*. Only data related to the core category *maintaining presence* were included. The interconnection of categories was established by identifying patterns and forming hypothetical relationships between them. This core process tied all the categories together and described how the HCPs strove to establish a trusting relationship as a prerequisite for existential conversations.

### Ethical considerations

Ethical approval for the study was attained from the Ethical Review Authority in Sweden (Dnr 2021–04117/2023-02950-02). Ethical considerations followed the research ethical rules presented in the Declaration of Helsinki. Approval was obtained from the managers at the different units where the study was conducted. The HCPs and the managers received oral and written information about the study and an invitation to participate. All participants gave their written informed consent before joining the study, and participants were given the option to withdraw their consent to participate at any time. Participants´ confidentiality was guaranteed, as was their anonymity in the presentation of the findings.

### Authors’ pre-understanding

The authors possess a certain pre-understanding as specialist nurses, hospital social workers, and researchers with experience encountering patients in PC and conducting conversations both as clinicians and as researchers with patients in different ages, partner, children and other family members together or individually. Therefore, continuous and ongoing discussions were conducted in the research group to counteract the influence of this pre-understanding and prevent it from biasing the results.

## Results

**Fig. 1:**
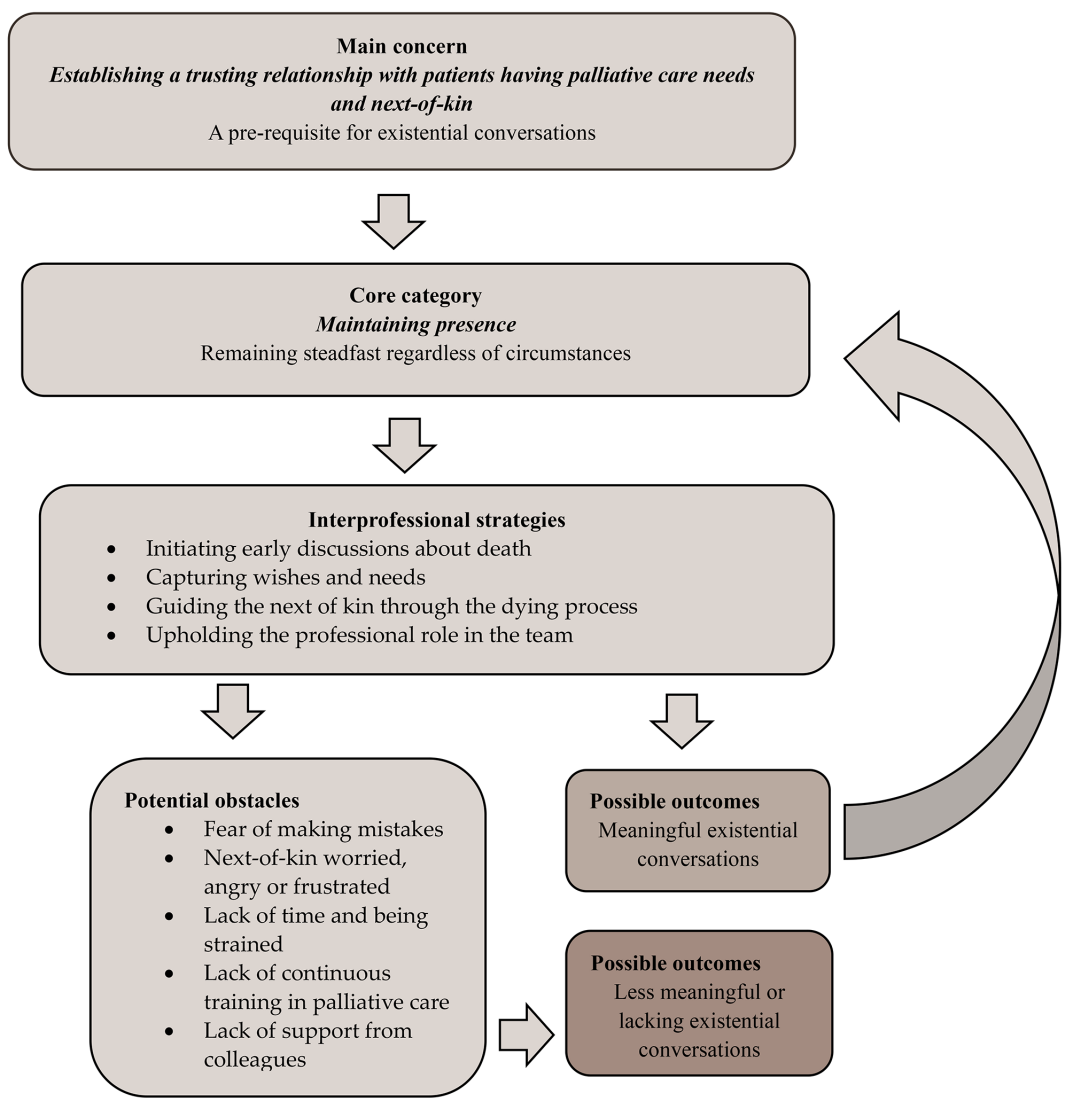
The emergent theoretical model for meaningful existential conversations in PC.

The results are supported by quotations described within brackets by type of profession and FG in the main category and potential hindrances sections. The core process describes how HCPs strove to *establish a trusting relationship* as a prerequisite for existential conversations in PC and how the core category of *maintaining presence* was central to every aspect of the communication process. The process involved professionals’ different challenges and strategies when engaging in these conversations. The following specific strategies were noted during the conversation process: *initiating early discussions about death* (starting conversations about thoughts concerning death), *capturing wishes and needs* (talking about memories and showing an interest in a patient’s life story), *guiding the next-of-kin through the dying process* (actively showing the next-of-kin how to participate in the care of the dying person and the dead body) and *upholding the professional role in the team* (contributing with specific skills, values and attitudes during existential conversations and collaborating with other disciplines). If a trusting relationship was not established due to potential obstacles, then meaningful existential conversations involving life, dying and death did not occur (Fig. 1).

### Establishing a trusting relationship– the primary concern

*Establishing a trusting relationship* is a prerequisite for engaging in existential conversations with severely ill patients and their next-of-kin, and it emerged as the main concern for the HCPs in PC. The HCPs were primarily concerned with establishing a relationship and strove to enable this using different strategies. They were attentive to patients’ existential concerns and made time to listen to their thoughts and requests. However, sometimes, they did not know how to meet expressions or were afraid to make mistakes when discussing life and death with a patient and their next-of-kin. In these instances, ANs would leave a brochure or call for the RN. Our results showed that a trustful connection was required to discuss sensitive and challenging concerns with patients and their next-of-kin. If trust was not established, it constituted a potential barrier to existential conversations and meaningful conversations about death and dying.

### Maintaining presence– the core category

*Maintaining presence* emerged as the core category (i.e. remaining steadfast regardless of circumstances). In encounters with patients and next-of-kin, HCPs stayed physically close by and were present in moments of silence. Staying nearby in daily care fostered a comforting and welcoming atmosphere. *Maintaining presence* also enabled existential conversations to take place that involved closure, sharing memories and supporting quality of life, even if the patient and next-of-kin were in a crisis or felt fear. When professionals upheld a state of being present, calm and friendly (as opposed to being strained), they perceived themselves as receptive to facilitating trusting, existential conversations.

### Main categories comprising interprofessional strategies

The resultant grounded theory comprised four inter-professional strategies: *initiating early discussions about death*,* capturing wishes and needs*, *guiding the next-of-kin through the dying process* and *upholding professional roles and collaboration*.

### Initiating early discussions about death

This strategy was based on the understanding that HCPs should initiate and conduct existential conversations about death as soon as a patient is admitted to a ward. This was achieved by openly inquiring about patients’ emotions and sensitively exploring their thoughts regarding the future and their hopes and fears. If the patient’s preferences were supported and expressed early in the illness trajectory, it allowed for the dissemination of knowledge to all involved professionals concerning the patient’s unique existential needs. For example, during initial conversations, professionals initiated discussions concerning the preferred place of death, i.e. if the patient desired to be transferred to a hospital near the end-of-life or remain at a nursing home. If the patient indicated a need to hold an existential conversation by sharing their thoughts on life and death, the professionals often endeavoured to pause and listen attentively.

‘*For the most part*,* I listened and listened present. It was their moment*,* and I existed for them. The most important thing*,* I think*,* is listening’* (RN, FGIV).

### Capturing wishes and needs

*Initiating early discussions about death* is a complex issue in PC, but it can be an entry point for enabling communication and facilitating the possibility to *capture the wishes and needs* of the patient. HCPs sought to establish trust and initiate existential conversations by demonstrating their availability, dedicating time and letting the patient and next-of-kin know they were open to discussions whenever desired. One strategy to *capture wishes and needs* was to talk about memories.

‘*We talked a little bit about what they had done when they were young and looked at photographs*,* and then we said: “If there is anything*,* we are here ‘…’ and if you want to talk*,* we’re here”’* (AN, FGV).

Sometimes, professionals received direct questions from a patient who wanted help to end their life in advance. In these instances, the professionals would meet these questions by initiating dialogue focused on the fact that no one could tell when death would come, and they would do everything to make the patient’s remaining time as comfortable as possible without shortening it. Another strategy was to provide medical information about their diagnosis and describe how symptoms can/will be alleviated if they become overwhelming.

### Guiding the next-of-kin through the dying process

During this process, professionals supervised the next-of-kin by engaging with their different questions, thoughts and feelings as their loved one approached death. In these situations, the next-of-kin could be worried, angry and/or frustrated. Thus, the HCPs aimed to support them by responding to their feelings and managing them with confidence and expertise. This involved not interpreting threats and criticism personally and by behaving calmly and acknowledging the concerns expressed by the next-of-kin. They also supervised the next-of-kin by providing clear and concrete information about potential future scenarios. For instance, when breathing became irregular, or if the next-of-kin wanted to connect an intravenous drip, they would explain the danger of giving an intravenous infusion to a dying person. HCPs took the initiative to prepare the next-of-kin by practically guiding and cooperating with them, i.e. by actively showing them how to participate in the care of a dying patient and taking care of the dead body, and by supporting and guiding them to say goodbye in peace (for example, by encouraging them to touch the patient).

‘*I’ve been on occasions where the next-of-kin… have never seen a dead person*,* and then they stand there helpless. So it becomes my job to guide them: “Yes*,* but maybe you should hold your mum’s hand”. “No*,* I don’t dare”*,* “No*,* but I can hold her for you”… and so on’* (RN, FGV).

### Upholding the professional role in the team

By *upholding the professional role in the team*, all HCPs contribute their specific competence, values and attitudes when communicating with patients and their next-of-kin. If a next-of-kin was upset and worried, the RNs would arrange a meeting in a secluded area on the care unit. However, PTs found it challenging to motivate patients to embrace living rather than merely existing (for instance, when a patient expressed that exercising was futile due to their impending death). According to the HCPs, availability and easy access to each other created a familiar and safe atmosphere that paved the way for existential conversations. For example, a strategy used by the PTs was to stay and talk about the patient’s previous activity habits.

‘*Meeting the ill person as a human and not just that I want the patients to get up and exercise’* (PT, FGII).

Existential conversations often occurred when talking to a patient about returning home and receiving home healthcare. The OTs found that during home visits, it often became obvious what kind of problems the patient had and what assistive devices they needed. These conversations could awaken many emotions and frustrations revolving around the patient’s circumstances in daily life.

‘*It´s a balancing act depending on which patient you meet and treat*,* and it is then important to get to know the ill person’* (OT, FGII).

One strategy was to collaborate with other professionals when they needed to reflect on demanding existential conversations. This depended on an understanding and openness in the team, where the professionals supported each other after having conversations about end-of-life care with patients and their next-of-kin. In particular, the RNs, ANs and CAs often felt alone when engaged in existential discussions. Furthermore, some RNs believed RNs, ANs and CAs as well as often lacked education and training and consequently lacked a PC approach in daily care or did not regard existential conversations as an integral part of PC. Hence, the RNs’ strategy was twofold: to supervise the ANs and CAs in PC and to ask for their help and support, as they typically had more extensive knowledge about the patient and their next-of-kin. RNs, Ans and CAs worked as an informal team and mutually benefited from one another’s competencies.

### Potential obstacles preventing existential conversations

Meaningful existential conversations with patients and their next-of-kin included aspects that HCPs perceived as hindrances, including *fear of making mistakes; next-of-kin’s worries*,* anger and frustration; lack of time and feeling strained; lack of training in PC;* and a *lack of support from colleagues*.

#### Fear of making mistakes

Fear of making mistakes may involve avoiding in-depth dialogue, emotional closeness, or physical contact.

‘*I think that as a staff member*,* I wouldn’t bring it up*,* but I think that it’s up to the next-of-kin and patients to bring it up if they want to talk about existential issues. I don’t think I would sit down and start talking about it if I don’t notice that the person wants to or sort of opens up to it’* (AN, FGV).

Fear of making mistakes also meant that the professionals did not always actively listen or be receptive to conversations by making eye contact or being attentive to the signals that the patient or next-of-kin communicated in the form of questions or cues. Limited hospital stays were another obstacle to building trusting relationships and getting close to patients and their next-of-kin.

#### Next-of-kin’s worries, anger and frustration

At times, the next-of-kin were perceived as aggressive towards HCPs, which was interpreted as being afraid that death was near and that the next-of-kin were about to lose their loved one. According to the HCPs, the next-of-kin sometimes expressed worry or anger that no one was at hand for the patient or feared that the patient would be left to suffer and die alone. If the family could not talk to each other about their fears or had conflicted relationships, this was seen by the professionals as an obstacle to letting the patient go and allowing them to die peacefully.

‘*Today*,* the next-of-kin don’t always want to let their mum*,* dad*,* grandma*,* grandpa*,* or anyone in the family pass away in peace. They want the ill family member to go to the hospital and do all the examinations imaginable…. even if you are 103 and a half years old; you should have done it….’* (RN, FGIV).

#### Lack of time and feeling strained

Existential conversations with severely ill patients or their next-of-kin were not always possible due to the HCP’s lack of adequate communication skills or time. When patients or next-of-kin initiated existential conversations, it was not always possible for the HCPs to respond immediately, as they were busy or involved with other challenging care situations. The professionals also found it stressful and time-consuming to engage in conversations with patients who had not yet come to terms with the reality of their death. Furthermore, HCPs found it challenging to communicate with next-of-kin who lacked acceptance that death was imminent for their ill family member. In these conversations, the communication could become complicated and stressful by denials or attitudes that ‘no one must die’ or a sense that there was a lack of trust in the HCPs.

‘*Two daughters said that if we didn’t send their mom to hospital*,* they would report us to the police. Yes*,* that conversation about end-of-life was very demanding’* (RN, FGV).

Feelings of stress were also created when the HCPs realised there was a conflict between the next-of-kin and the patient. In these situations, the professionals were negatively affected emotionally.

#### Lack of continuous training in PC

Addressing existential concerns in conversations with patients was not universally perceived as an integrated and seamless aspect of caring across different contexts. Frequently, HCPs felt uncertain about how to talk to severely ill patients who expressed hopelessness and meaninglessness at the end-of-life. The RNs perceived the entire team to lack continuous training in existential conversations in PC.

‘… *when you are new*,* i.e. new employees*,* there should be more training there directly in this [existential dialogue] than that you should learn the first time you stand there….’* (RN, FGVII).

Colleagues whose perspectives differed from those of the patient and next-of-kin due to cultural differences or a ​​lack of strategies were challenging. This meant that HCPs who avoided talking about death and dying with patients or next-of-kin did not conduct any existential conversations. To learn and better respond and converse with patients and next-of-kin about existential issues, the HCPs expressed a need for continuous training. They also lacked training in basic conversation techniques, conducting conversations with people in crisis and grief and handling existential issues. Furthermore, the HCPs did not have access to continuous joint team supervision. While some professions such as physicians and hospital social workers had separate supervision sessions, RNs and ANs did not. Education and training in communication are crucial:

‘*[To learn about communication] it is important to make it as dignified and nice as possible when next-of-kin come… if they choose to come and say goodbye.’* (RN, FGVI*).*

#### Lack of support from colleagues

At times, HCPs felt there was a lack of support from the rest of the team and felt alone in conducting existential conversations with patients and next-of-kin, while temporary workers would ask someone else on the team to conduct existential conversations:

‘*… if you work with temporary workers*,* they ask you: “Can you go and talk to the next-of-kin” because they may not know the patient as I do*,* so then I become the main person responsible for talking to the next-of-kin and so on’* (RN, FGVI).

The physicians were considered important for explaining medical issues and being part of existential conversations, but they did not always accept that responsibility. Instead, that rested primarily on RNs and ANs who lack the medical and professional mandate of physicians. The HCPs described different professions as having different roles in PC. For example, it was common for the ANs to refer to the RNs if they could not answer or handle questions from patients or next-of-kin. However, the HCPs were, for the most part, uncertain about how the healthcare organisation could assist regarding professional support. Consequently, they experienced a sense of being left to handle much on their own.

### The emergent theoretical model for meaningful existential conversations in PC

The proposed model was developed from the data and captured the process of *establishing a trusting and dignified relationship as a prerequisite for existential conversations in palliative care*. The core category of *maintaining presence* was central to every aspect of the communication process (Fig. 1). The process involved the different challenges and strategies professionals faced in existential conversations. Specific strategies were distinguished during the conversation process including: *initiating early discussions about death* (starting conversations on thoughts about death early), *capturing wishes and needs* (talking about memories and showing an interest in a patient’s life story), *guiding the next-of-kin through the dying process* (actively showing next-of-kin how to participate in the care of the dying person and the dead body) and *upholding the professional role in the team* (contributing with specific skills, values and attitudes in existential conversations, and collaborating with other disciplines). This could be going on in a process leading to new existential conversations, as long as the four inter-professional strategies to maintain the presence were activated. Certain hindrances were also noted throughout the conversation process, including: *fear of making mistakes* (not being attentive to the patient’s signs), *next-of-kin’s worries*,* anger and frustration* (when the next-of-kin feel that no one is available for their ill family member), *lack of time and feeling strained*, (engaged in many different, challenging situations), *lack of continuous training in PC* (needing supplementary education about existential conversations) and *lack of support from colleagues* (feeling alone in carrying out existential discussions). If a trusting and dignified relationship was not established due to potential hindrances, meaningful existential conversations involving issues of life, dying and death did not occur.

## Discussion

This study aimed to gain an in-depth understanding of HCPs’ experiences of existential conversations with patients with PC needs and their next-of-kin by generating a theoretical model. This model illustrates how HCPs in general and in specialised PC situations strive to establish meaningful existential discussions. Establishing a trusting relationship was found to be a prerequisite for meaningful existential conversations to occur. However, barriers were also identified that can hinder existential discussions to the point where they do not happen or are less meaningful. This study brings information together in a model and provides information on the strategies HCPs use to overcome obstacles. According to Fredriksson and Eriksson [20], the conversational context involves the ontology of the severely ill patient’s life world and knowledge about their experiences of suffering. In our study, HCPs used strategies of *capturing wishes and needs* to support patients and help them express their suffering and confront it. Previous studies have reported facilitating factors for conversations about end-of-life care, such as structural variables, time, setting, availability and willingness to discuss end-of-life issues [21, 22]. We also identified potential barriers to meaningful existential conversations, including fear of making mistakes, emotions such as anger and frustration, time constraints, feelings of stress, inadequate training in PC, insufficient integration of PC within the care context and a sense that there is a lack of support from colleagues.

Communication is a cornerstone of PC; nevertheless, barriers may hinder meaningful existential conversations and quality of life for patients close to death. This was also reported in a study by Strang et al. [23]. They found that existential conversations sometimes felt burdensome to HCPs, which could lead to a lack of such discussions. As Klarare et al. [7] highlighted, effective inter-professional communication, whether among team members or between HCPs and patients, is a prerequisite for fostering trust and providing support.

The present study offers insight into several interdisciplinary strategies HCPs used to establish a trusting relationship and initiate existential conversations early on. First, when a severely ill patient is enrolled in a PC unit, conversations about the patient’s values and thoughts about imminent death should take place. The effect of early conversations can also be extended to include the next-of-kin, who can support the patient, help them to make the most of the time they have left and allow them to prepare for the patient’s death while also maintaining hope [22, 24]. Consistent with these findings, De Panfilis et al. [25] suggested that caring for severely ill patients involves entering into a demanding and difficult relationship. Stenman et al. [26] described how the caring relationship and conversations in PC frequently entail confidentiality and include existential challenges. Integral parts of meaningful, continuous, existential conversations in our study were active listening and not always using words to communicate but using body language and eye contact. Stenman et al. [26] also found that listening was a responsibility of RNs in PC conversations, with the aim to avoid distressing the patient.

Another strategy to manage existential conversations was to appreciate the patient’s wishes, values and needs by showing an interest in their life stories and seeing the person behind the illness. Fredriksson and Eriksson [20] considered it crucial that patients talk about their well-being or suffering with others to regain their self-esteem and self-determination. According to Stephen et al. [21], patients also need sufficient time to discuss existential issues to better manage other difficult matters near the end-of-life, and, to that end, HCPs should be available to the patient and the next-of-kin. In our study, HCPs guided patients and the next-of-kin through the dying process even though they were, at times, insecure about what to say due to a lack of PC and communication training. Furthermore, in nursing, person-centred care involves tailoring care to a person’s specific needs and problems, which may include an assessment of their physical symptoms, counselling and emotional support related to the illness, treatment and care [27]. However, the delivery of PC, including existential conversations about death and dying, is complex and involves a team of different HCPs. According to Spruyt [28], creating an effective multidisciplinary team with HCPs is one of the greatest challenges in PC. The HCPs in our study attempted to uphold a professional role in the multidisciplinary team by talking to other professionals when they had a demanding existential conversation with dying patients and their next-of-kin. According to De Panfilis et al. [25], professionals are advised to provide mutual support and assume accountability for their professional roles and ethical commitments. This entails comprehending the significance of ethical considerations within care relationships by respecting the patient’s dignity and values.

In the present study, the RNs, ANs and CAs at nursing homes reported a lack of continuous training in PC as a barrier to conducting meaningful existential conversations. This is not surprising since the professionals were working in contexts where general PC were provided, not specialized PC, which is important to keep in mind. However, existential conversations can be expected also in other care than palliative. While some HCPs considered existential conversations an integral part of their professional practice, others did not. Other studies have also reported a lack of PC education and involvement in decision-making among HCPs at nursing homes [29, 30]. In Sweden, education and training concerning palliative communication among HCPs varies across care units and regions and between different professions. RNs and physicians typically receive more extensive education than other HCPs [5], including ANs. These differences may lead to a lack of existential conversations with patients with PC needs and their next-of-kin. Previous studies have reported the successful use of different team activities such as consultations, team meetings and debriefings to sustain and support its members. Thus, generosity and effective communication between team members are vital to achieving successful team outcomes that improve end-of-life care for patients and their next-of-kin [28, 31].

### Methodological considerations

There are a number of strengths in the present study. First, various HCPs from different contexts spoke openly about their experiences in existential conversations with PC patients. Second, both rural and urban regions in Sweden were represented in the study. Third, the interactions between HCPs in the FGs produced data on topics that were not explicitly asked for, such as how to prepare the next-of-kin by guiding and showing them how to participate in the practical care of their dying relative and, later, the dead body. Finally, verbatim quotations were used when presenting the results to elucidate and validate the grounding of the data and strengthen its trustworthiness. The quality of the GTM should be assessed based on the criteria’s ‘fit,’ ‘work,’ ‘relevance’ and ‘modifiability’ [32]. The most important issue to ensure trustworthiness in our study was applying the GTM correctly. The theoretical model emerged from empirical findings and fit the context of the study. Moreover, the model can be a useful guiding tool in PC as it explains some of the strategies for and obstacles to accomplishing a meaningful conversation about existential issues in PC. The model’s relevance is indicated by how it reflects the daily concerns that occupy HCPs working in PC. In conclusion, the model is adaptable to other care contexts and is open for further development.

### Limitations

Gender representation among HCPs was unequal (females, *n* = 19, male *n* = 3). However, this inequality provides an accurate picture of the gender distribution among HCPs in Sweden. There were few OTs and PTs in our study, but those who did participate described having existential conversations with dying patients. In PC, they have an advisory role and specialised knowledge of palliative rehabilitation and how to enable patients to maintain meaningful activities. However, they often learn too little or too late about patients at the end-of -life. Therefore, the existential conversation processes described in this study come mainly from the perspective of ANs and RNs in PC, who shared their experiences initiating and managing existential conversations. However, RNs and ANs are the ones that are close to and most frequently care for and communicate with severely ill and dying patients and next-of -kin and were able to provide rich information according to the aim of this study. Finally, the situations described from the perspective of HCPs are subjective and retrospective.

## Conclusion

This study illuminated HCP’s experiences of existential conversations with severely ill and dying patients with PC needs and their next-of -kin. It illustrates the main concerns and interdisciplinary strategies used by HCPs in various PC contexts when establishing a trusting relationship with patients and their next -of- kin. This may guide other professionals in a broad range of PC contexts. The constructed theoretical model in this study emphasises strategies HCPs use when enabling meaningful existential conversations that involve life, dying and death in PC. The potential obstacles we identified that require attention include the need for managers of nursing homes and specialist PC units to support HCPs by allowing time for reflection, developing new routines, and providing education focused on existential conversations in PC. However, the main contribution of this study is the description of the interdisciplinary strategies HCPs used to establish meaningful, existential conversations by maintaining presence, as this paved the way for trusting conversations with patients and next-of-kin. The model can be used as a basis for reflection in collaborative learning interventions within PC and as a tool for teachers in educational PC programmes. The model is planned to be validated and tested in educational interventions with HCPs in different healthcare contexts.

## Electronic supplementary material

Below is the link to the electronic supplementary material.

Supplementary Material 1

## Data Availability

No datasets were generated or analysed during the current study.
