# Supplementary material for: Interdisciplinary strategies for establishing a trusting relation as a pre-requisite for existential conversations in palliative care: a grounded theory study
Source: BMC Palliat Care. 2025 Feb 19;24:47. doi: 10.1186/s12904-025-01681-x (PMC11837293; doi:10.1186/s12904-025-01681-x)
Supplement: Supplementary file 1 — Supplementary Material 1 [file 12904_2025_1681_MOESM1_ESM.docx]

Interviewguide in focusgroups with different healthcare professionals' on conversations with patients with palliative care needs

**Data collection phase I**

Main questions:

Can you tell me what conversations arise when you are caring for patients and when meeting their next-of-kin?

Can you describe what make a conversation good or less good?

Follow-up questions:

What do the patients and next-of-kin bring up?

What facilitates a conversation?

What makes a conversation difficult?

How safe do you feel in situations where difficult conversations take place?

Is there anything you want to add that we haven't talked about?

**Data collection phase 2**

Main questions:

Can you tell me how you communicate with a patient regarding their thoughts about death?

Can you describe how you respond to patients’ existential questions?

Can you tell me how you respond to next-of-kin who don´t accept the situation?

Follow-up questions:

How do you open up for conversations about existential issues?

How do you handle that situation if the patient brings it up themselves?

How do you create trust/confidence in the relationship?

How are next-of-kin included in conversations about existential issues?

How do you feel about these conversations (difficult- don't want to, don't have time/ great- now I make time)?

Is there anything you want to add that we haven't talked about?
